# Three-dimensional deep learning model complements existing models for preoperative disease-free survival prediction in localized clear cell renal cell carcinoma: a multicenter retrospective cohort study

**DOI:** 10.1097/JS9.0000000000001808

**Published:** 2024-06-19

**Authors:** Yingjie Xv, Zongjie Wei, Qing Jiang, Xuan Zhang, Yong Chen, Bangxin Xiao, Siwen Yin, Zongyu Xia, Ming Qiu, Yang Li, Hao Tan, Mingzhao Xiao

**Affiliations:** a Department of Urology, The First Affiliated Hospital of Chongqing Medical University; b Department of Urology, The Second Affiliated Hospital of Chongqing Medical University; c Department of Urology, The Affiliated Yongchuan Hospital of Chongqing Medical University; d Department of Urology, Chongqing University Fuling Hospital; e Department of Urology, Chongqing University Three Gorges Hospital; f Department of Urology, The People’s Hospital of Dazu, Chongqing, People’s Republic of China

**Keywords:** computed tomography, deep learning, disease-free survival, localized ccRCC, radiomics

## Abstract

**Background:**

Current prognostic models have limited predictive abilities for the growing number of localized (stage I–III) ccRCCs. It is, therefore, crucial to explore novel preoperative recurrence prediction models to accurately stratify patients and optimize clinical decisions. The purpose of this study was to develop and externally validate a computed tomography (CT)-based deep learning (DL) model for presurgical disease-free survival (DFS) prediction.

**Methods:**

Patients with localized ccRCC were retrospectively enrolled from six independent medical centers. Three-dimensional (3D) tumor regions from CT images were utilized as input to architect a ResNet 50 model, which outputted DL computed risk score (DLCR) of each patient for DFS prediction later. The predictive performance of DLCR was assessed and compared to the radiomics model (Rad-Score), the clinical model the authors built and two existing prognostic models (UISS and Leibovich). The complementary value of DLCR to the UISS, Leibovich, as well as Rad-Score were evaluated by stratified analysis.

**Results:**

Seven hundred seven patients with localized ccRCC were finally enrolled for models’ training and validating. The DLCR the authors established can perfectly stratify patients into low-risks, intermediate-risks, and high-risks, and outperformed the Rad-Score, clinical model, UISS and Leibovich score in DFS prediction, with a C-index of 0.754 (0.689–0.821) in the external testing set. Furthermore, the DLCR presented excellent risk stratification capacity in subgroups defined by almost all clinic-pathological features. Moreover, patients classified as low-risk by the UISS/Leibovich score/Rad-Score but as intermediate - or high-risk by DLCR were significantly more likely to experience ccRCC recurrence than those stratified as intermediate- or high-risk by UISS/Leibovich score/Rad-Score but as low-risk by DLCR (all Log-rank *P-*values<0.05).

**Conclusions:**

Our DL model, derived from preoperative CT, is superior to radiomics and current models in precisely DFS predicting of localized ccRCC, and can provide complementary values to them, which may assist more informed clinical decisions and adjuvant therapies adoptions.

## Introduction

Highlights

- Three-dimensional (3D) deep learning (DL) model enabled accurate preoperative disease-free survival prediction in patients with localized clear cell renal cell carcinoma.
- 3D DL model outperformed the radiomics model, clinical model, as well as two existing models (UISS and Leibovich).
- The established DL computed risk score provided complementary values to existing traditional models and the Rad-Score.

In the US, there were 81 800 new cases of renal cell carcinoma (RCC) diagnosed in 2023, resulting in 14 890 deaths^1^. Clear cell renal cell carcinoma (ccRCC) is the most common subtype of RCC that bears the highest mortality rate^2^. Although curative surgery is effective in the majority of localized (stage I–III) cases of ccRCC, up to 20–30% of patients are still at risk of disease progression^3^. Research indicates that postoperative adjuvant therapies, such as immunotherapy and targeted interventions, could benefit localized ccRCC patients at high risk^4,5^. It is, therefore, crucial to diligently monitor the preoperative prognosis of patients with localized ccRCC for personalized clinical decision-making.

Conventional prognostic factors, including TNM classification and histopathological nuclear grade, are useful in predicting RCC prognosis^6,7^. Nevertheless, due to the clinical heterogeneity of patients at the same stage, their clinical usefulness remains limited^7–9^. For patients with localized ccRCC, the 2003 Leibovich score and the University of California, Los Angeles, Integrated Staging System (UISS) are two commonly used prognostic models with more robust performance, due to their integration of multiple prognostic factors^10–13^. However, both models were developed long since, and updated versions of their key factors have been introduced, resulting in uncertain clinical utilities for current patient populations. Additionally, both models lack the incorporation of molecular indicators that consider tumor heterogeneity, highlighting the need for improvements in predictive accuracy.

As part of the routine preoperative examination for RCC patients, a computed tomography (CT) scan offers informative anatomical prognostic details as well as essential tumor heterogeneity-related prognostic features that are frequently disregarded^14,15^. Artificial intelligence (AI), specifically radiomics and deep learning (DL), provides novel approaches to extracting and analyzing molecular biology data on tumor heterogeneity from medical images^16–19^. Earlier studies have illustrated that radiomics features derived from CT images are associated with patients’ prognosis across various tumor types, including ccRCC^20–24^. The prognostic model they built revealed commendable predictive ability, evidenced by C-indexes ranging between 0.630 and 0.704 in the validation sets. The use of DL algorithms has enabled automated, comprehensive analysis of medical images for multitasks, such as tumor prognosis^25–27^. For instance, employing multiple DL architectures, Jiang *et al*.^28^ developed and compared prognostic models based on preoperative MRI for survival prediction of patients with rectal cancer. The ViT model they built yielded a C-index of 0.69 for disease-free survival (DFS). Hao *et al*.^29^ proposed a novel ‘End-to-End’ DL architecture to predict progression-free survival in patients with gastric cancer, with a resulting C-index of 0.783 in the validation set. However, as far as we know, there is still a lack of DL model for preoperatively predicting DFS of localized ccRCC patients. Besides, no conclusive evidence demonstrating the superiority of the CT image-based DL model over the manually dependent radiomics model in the prognostic prediction of patients with localized ccRCC. Moreover, there have been few studies that compare established models with existing conventional prognostic systems and explore the complementary values of these models.

This study aimed to develop and externally validate a DL computed risk score (DLCR) for DFS prediction in patients with localized ccRCC, based on 3D tumor regions from preoperative contrast-enhanced CT scans. The predictive performances of various models, including the DL model, radiomics model, clinical model, and existing traditional models, were compared. Additionally, the complementary value of DLCR to UISS, Leibovich score, and radiomics model were explored. Furthermore, a combined model was promoted and visualized via a nomogram, to aid the clinical application value.

## Methods

### Patients

**Figure 1:**
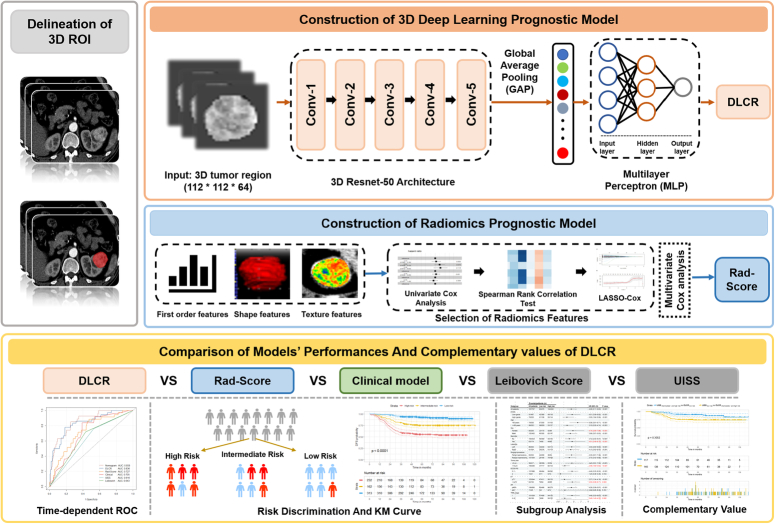
Overview of the study workflow.

This retrospective multicenter study enrolled patients from six independent Chinese hospitals. The ethic approval was obtained from the institutional review board and the patients’ informed consent was waived (2022-KY508). The study was registered on the ClinicalTrials.gov network. The research design and paper writing were in accordance with the strengthening the reporting of cohort, cross-sectional, and case–control studies in surgery (STROCSS) (Supplemental Digital Content 1, http://links.lww.com/JS9/C771) criteria^30^ and the Declaration of Helsinki. The overall workflow of this study is illustrated in Figure 1.

The same inclusive and exclusive criteria were applied in the six centers. Patients who underwent partial/radical nephrectomies and histologically diagnosed as ccRCC were initially enrolled. We excluded patients: (1) with incomplete clinic-pathological data; (2) lack of preoperative contrast-enhanced CT images or the image quality was unsuitable for analysis; (3) who received presurgery neoadjuvant or adjuvant therapies; (4) with multiple renal tumors or/and had synchronous metastasis. The detailed patients’ recruitment and data split progression are presented in Supplementary Fig. S1 (Supplemental Digital Content 2, http://links.lww.com/JS9/C772). Patients were enrolled during two time periods: (1) At Center 1, Center 2, and Center 3, recruitment occurred between December 2013 and March 2020; (2) At Center 4, Center 5, and Center 6, recruitment took place between June 2013 and December 2016. A total of 707 patients were finally recruited. Patients derived from the Center 1 (*n*=314), Center 2 (*n*=54), and Center 3 (*n*=152) were randomly assigned to the training (*n*=364), and internal testing (*n*=156) sets with a split ratio of 7:3, while the external testing set (*n*=187) consisted of patients from the Center 4 (*n*=89), Center 5 (*n*=75), and Center 6 (*n*=23).

### Follow-up and clinic-pathological data

The follow-up outcome measure was DFS, denoting the interval from the date of surgery to disease recurrence, all-cause mortality or the last visit. The postoperative follow-ups were conducted every 6 months for the first 3 years and then annually via electronic medical records (EMRs) and telephone enquiries. The follow-up endpoint was 10 March 2023.

Clinic-pathological information such as sex, age, tumor laterality, surgery procedure, Fuhrman grade, tumor size, Eastern Cooperative Oncology Group Performance Status (ECOG-PS), pathological T stage, pathological N status, and pathological necrosis were collected. The TNM stage was evaluated according to the guidelines from the American Joint Committee on Cancer ‘Cancer Staging Manual, eighth edition’^31^. The evaluation methods of Leibovich^11,32^ and UISS^32^ are summarized in Supplementary S1 (Supplemental Digital Content 2, http://links.lww.com/JS9/C772).

### CT acquisition and tumor region delineation

All included patients underwent abdominal CT examinations within 2 weeks before surgery. The Picture Archiving and Communication Systems (PACS) were used to download the original Digital Imaging and Communications in Medicine (DICOM) format CT images. The corticomedullary phase (CMP) images were utilized for the ensuing analysis in this study. The parameters for CT scanners and definitions of various CT phases are listed in Table S1 and Supplementary S2 (Supplemental Digital Content 2, http://links.lww.com/JS9/C772). Manually delineated three-dimensional (3D) region-of-interests (ROIs) were conducted by two radiologists (Reader A and B; both with over 10-year urological imaging reading experiences) who were blinded to patients’ clinic-pathological information via the ITK-SNAP software (Version 3.8, http://www.itksnap.org/pmwiki/pmwiki.php). The segmented ROIs were checked by a third senior radiologist (Reader C), who has more than 15 years of experience in image processing for renal cancer. Reader A primarily segmented all images. Two weeks later, 70 patients were randomly selected and remarked by reader A and reader B to calculate intraobserver and interobserver intraclass correlation coefficients (ICCs). The repeatability of segmentations was evaluated via ICCs.

### Establishment of deep learning model

The segmented 3D tumor region of each patient was chosen as the input data. The CT images were filtered with a window of [−30, 300] HU and resized to 112×112×64 voxels. The pixel values were normalized to [0–1]. Data augmentation methods, including random clipping, rotation and flipping were applied in the training set to improve the model’s generalization performance.

Three 3D DL models (3D ResNet-50, ResNet-18, and ResNet-101) were utilized as the backbone network architecture to extract prognostic image features from 3D tumor regions. The 3D ResNet-50 architecture outperformed ResNet-101 and ResNet-18, therefore was used as the final backbone network in the convolutional module (Supplementary Table S2, Supplemental Digital Content 2, http://links.lww.com/JS9/C772). A linear layer was connected to initially trained it for obtaining the feature extractor. A global average pooling layer was incorporated into the final convolutional layer to dig the in-depth feature maps^33^. This approach provides several advantages, notably establishing meaningful correlations between feature maps and classes and enhancing interpretability within convolutional structures. Additionally, the process of parameter elimination facilitates optimization, acting as a preventative measure against overfitting. Subsequently, a prognostic risk calculation module was constructed using a series of layers in a multilayer perceptron (MLP) configuration. The MLP included a hidden layer with 256 neurons, an activation function layer (ReLU), and a batch normalization layer. The convolutional network was initially frozen, allowing the MLP to be trained to integrate high-dimensional features effectively. Following this, all parameters of the network were unfrozen for an end-to-end training phase, utilizing a smaller learning rate. Finally, the DLCR of each patient was yielded by the final output layer, which consisted of a single neuron.

A Nvidia GeForce RTX 3090 GPU with the Pytorch framework was utilized to the model training. The loss function based on Cox partial likelihood was applied to evaluate the survival differences between patients during optimization^34^. The feature extraction network and prognostic risk calculation network were initialized with an initial learning rate of 1e-4, whereas the end-to-end fine-tuning phase commenced with an initial learning rate of 1e-6. Subsequently, during the fine-tuning process, the learning rate was subject to a halving mechanism triggered when there was no observable enhancement in the C-index of the internal testing set for ten consecutive evaluations. The model underwent a maximum of 50 epochs of training, utilizing the Adam optimizer with a batch size of 32. Early stopping was implemented with a patience of 20. The hyperparameters for the model with the highest internal validation C-index were ultimately selected.

### Construction of radiomics model, clinical model, and combined nomogram

To ensure the comparability across different cases and CT scanners, image preprocessing, including resampling and gray-level discretization, was firstly conducted. The open source ‘Pyradiomics’ was used to extract radiomics features (Supplementary S3, Supplemental Digital Content 2, http://links.lww.com/JS9/C772), which were then screened via z-scores normalization, ICC, univariate Cox proportional hazards regression, Spearman rank correlation test, and least absolute shrinkage and selection operator (LASSO) Cox regression algorithm, orderly. Detailed radiomics feature dimension reduction procedures are summarized in Supplementary S4 (Supplemental Digital Content 2, http://links.lww.com/JS9/C772). The selected radiomics features were finally analyzed by a Multivariate Cox proportional hazards regression and the radiomics score (Rad-Score) of each patient was calculated accordingly.

Clinic-pathological characteristics were investigated by Univariate and Multivariate Cox proportional hazards regression, and the yielded prognostic factors were utilized to build a clinical model. Integrating with clinic-pathological features, Rad-Score, and DLCR, a combined nomogram was established. The prognostic abilities of UISS and Leibovich models were validated basing on the multicenter data.

### Performance evaluation and models’ comparison

The DFS predictive performances of models were assessed by the Harrell’s consistency index (C-index) and time-dependent receiver operating characteristic (ROC) curve analysis. Using X-tile software (https://x-tile.software.informer.com), the optimal cut-off values of DLCR and Rad-Score in the training set were computed and applied to stratify patients in other sets. The DFS differences between the stratified groups and subgroups split by pathological grade, age, sex, pathological necrosis, tumor laterality, surgical procedure, tumor size, ECOG-PS, pT stage, pN status, TNM stage, UISS risk and SSIGN risk were further explored by the Univariate Cox analysis, and Kaplan–Meier survival analysis with Log-rank test. The calibration curve analysis was performed to determine the consistency between nomogram predicted and observed DFS probability.

### Statistically analysis

Continuous variables with non-normal distributions were presented as medians (interquartile ranges) and analyzed using the Kruskal–Wallis test. The categorical variables (*n*, %) were compared using the *χ* ^2^ test or Fisher exact test, as proper. A two-tailed *P*-value <0.05 was considered as statistically significant. All statistical analysis was performed on R (version 4.3.1; https://www.r-project.org) and SPSS (version 23.0; SPSS Inc.) software.

## Results

### Clinic-pathological characteristics

The baseline clinic-pathological characteristics of the patients are presented in Table 1. A total of 707 patients were enrolled, comprising 452 males (63.9%) and 255 females (36.1%), with a median age of 59.0 years [interquartile range (IQR): 39.0–78.0] and a median tumor size of 4.4 cm (IQR: 1.8–10.0 cm). The overall median follow-up duration was 57.8 months (IQR: 40.6–83.3). Except for sex (*P*=0.001), Fuhrman grade (*P*=0.008), and follow-up time (*P*<0.001), there was no significant difference among training, internal testing, and external testing sets.

**Table 1:** Baseline clinic-pathological characteristics in the training and external testing sets.

Variable	All patients (*n*=707)	Training set (*n*=364)	Internal testing set (*n*=156)	External testing set (*n*=187)	*P*
Age					0.920
＜60 years	355 (50.2)	184 (50.5)	76 (48.7)	95 (50.8)	
≥60 years	352 (49.8)	180 (49.5)	80 (51.3)	92 (49.2)	
Sex					0.001^a^
Female	255 (36.1)	108 (29.7)	72 (46.2)	75 (40.1)	
Male	452 (63.9)	256 (70.3)	84 (53.8)	112 (59.9)	
Tumor laterality					0.560
Left	353 (49.9)	187 (51.4)	72 (46.2)	94 (50.3)	
Right	354 (50.1)	177 (48.6)	84 (53.8)	93 (49.7)	
Surgery procedure					0.446
Partial nephrectomy	353 (49.9)	188 (51.6)	79 (50.6)	86 (46.0)	
Radical nephrectomy	354 (50.1)	176 (48.4)	77 (49.4)	101 (54.0)	
Tumor size					0.250
＜5 cm	417 (59.0)	219 (60.2)	97 (62.2)	101 (54.0)	
≥5 cm	290 (41.0)	145 (39.8)	59 (37.8)	86 (46.0)	
Pathological necrosis					
Absent	533 (75.4)	274 (75.3)	125 (80.1)	134 (71.7)	0.195
Present	174 (24.6)	90 (24.7)	31 (19.9)	53 (28.3)	
Fuhrman grade					0.008^a^
I	180 (25.5)	95 (26.1)	48 (30.8)	37 (19.8)	
II	310 (43.8)	175 (48.1)	62 (39.7)	73 (39.0)	
III	186 (26.3)	80 (22.0)	41 (26.3)	65 (34.8)	
IV	31 (4.4)	14 (3.8)	5 (3.2)	12 (6.4)	
pT stage					0.780
T1a	311 (44.0)	156 (42.9)	76 (48.7)	79 (42.2)	
T1b	233 (33.0)	127 (34.9)	45 (28.8)	61 (32.6)	
T2	74 (10.5)	38 (10.4)	14 (9.0)	22 (11.8)	
T3	89 (12.6)	43 (11.8)	21 (13.5)	25 (13.4)	
pN status					0.847
N0/Nx	685 (96.9)	353 (97.0)	152 (97.4)	180 (96.3)	
N1	22 (3.1)	11 (3.0)	4 (2.6)	7 (3.7)	
TNM stage					0.931
I	539 (76.2)	280 (76.9)	120 (76.9)	139 (74.3)	
II	68 (9.6)	35 (9.6)	13 (8.3)	20 (10.7)	
III	100 (14.2)	49 (13.5)	23 (14.7)	28 (15.0)	
ECOG-PS					0.240
0-1	686 (97.0)	356 (97.8)	152 (97.4)	178 (95.2)	
2	21 (3.0)	8 (2.2)	4 (2.6)	9 (4.8)	
UISS risk					0.440
Low-risk	341 (48.2)	181 (49.7)	78 (50.0)	82 (43.9)	
Intermediate-risk	318 (45.0)	157 (43.1)	66 (42.3)	95 (50.8)	
High-risk	48 (6.8)	26 (7.1)	12 (7.7)	10 (2.3)	
Leibovich risk					0.291
Low-risk	413 (58.4)	219 (60.2)	96 (61.5)	98 (52.4)	
Intermediate-risk	217 (30.7)	109 (29.9)	41 (26.3)	67 (35.8)	
High-risk	77 (10.9)	36 (9.9)	19 (12.2)	22 (11.8)	
Follow-up (months)	57.8 (40.6–83.3)	54.0 (39.6–66.9)	58.2 (29.9–85.9)	79.9 (54.4–96.9)	<0.001^a^

a *P*<0.05.

### Development and external validation of DLCR

A DL model was successfully established for DFS prediction of patients with localized ccRCC in the training set, and the DLCR of each patient was calculated. The DLCR exhibited excellent DFS predicting performance, with C-index values of 0.804 [95% CI: 0.760–0.849), 0.781 (95% CI: 0.728–0.833), and 0.754 (95% CI: 0.689–0.821) in the training, internal testing, and external testing sets, respectively (Table 2).

**Table 2:** Predictive performances of various models for disease-free survival (DFS) of patients with localized ccRCC in the training, internal testing, and external testing sets.

Model	Cohort	C-index (95% CI)	3-year AUC (95% CI)	5-year AUC (95% CI)	7-year AUC (95% CI)
UISS	Training set	0.601 (0.544–0.658)	0.586 (0.519–0.652)	0.603 (0.534–0.672)	0.572 (0.479–0.666)
	Internal testing set	0.611 (0.531–0.691)	0.609 (0.525–0.693)	0.619 (0.529–0.709)	0.641 (0.541–0.741)
	External testing set	0.623 (0.553–0.694)	0.616 (0.524–0.709)	0.600 (0.515–0.685)	0.634 (0.550–0.718)
Leibovich	Training set	0.630 (0.573–0.687)	0.653 (0.588–0.719)	0.615 (0.544–0.686)	0.598 (0.504–0.693)
	Internal testing set	0.609 (0.535–0.683)	0.611 (0.525–0.696)	0.608 (0.517–0.699)	0.593 (0.490–0.697)
	External testing set	0.663 (0.539–0.786)	0.643 (0.546–0.739)	0.667 (0.584–0.751)	0.726 (0.647–0.805)
Clinical	Training set	0.663 (0.605–0.722)	0.683 (0.612–0.754)	0.650 (0.574–0.727)	0.629 (0.519–0.738)
	Internal testing set	0.683 (0.603–0.763)	0.705 (0.617–0.794)	0.702 (0.606–0.798)	0.664 (0.551–0.777)
	External testing set	0.665 (0.597–0.734)	0.705 (0.611–0.800)	0.703 (0.614–0.792)	0.734 (0.642–0.827)
Rad-Score	Training set	0.703 (0.647–0.759)	0.718 (0.649–0.787)	0.688 (0.612–0.764)	0.662 (0.558–0.767)
	Internal testing set	0.686 (0.614–0.758)	0.700 (0.610–0.790)	0.697 (0.601–0.794)	0.737 (0.633–0.841)
	External testing set	0.689 (0.617–0.760)	0.727 (0.639–0.815)	0.712 (0.627–0.796)	0.715 (0.625–0.804)
DLCR	Training set	0.804 (0.760–0.849)	0.848 (0.798–0.898)	0.854 (0.800–0.908)	0.830 (0.754–0.906)
	Internal testing set	0.781 (0.728–0.833)	0.787 (0.710–0.864)	0.852 (0.782–0.921)	0.913 (0.848–0.977)
	External testing set	0.754 (0.689–0.821)	0.836 (0.762–0.910)	0.797 (0.723–0.871)	0.806 (0.732–0.881)
Nomogram	Training set	0.829 (0.791–0.868)	0.866 (0.822–0.911)	0.873 (0.826–0.920)	0.862 (0.795–0.929)
	Internal testing set	0.795 (0.744–0.847)	0.832 (0.767–0.896)	0.828 (0.756–0.901)	0.899 (0.829–0.970)
	External testing set	0.803 (0.750–0.855)	0.858 (0.789–0.927)	0.871 (0.811–0.931)	0.869 (0.801–0.937)

CI, confidence interval.

**Figure 2:**
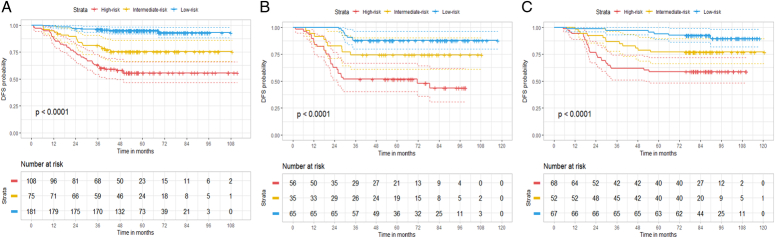
The Kaplan–Meier survival curves for disease-free survival (DFS) of localized clear cell renal cell carcinoma (ccRCC) patients, categorized by the deep learning computed risk score (DLCR), were analyzed in the training set (A), internal testing (B), and external testing (C) sets. The *P*-value was calculated using the log-rank test.

Based on the optimal DLCR cut-off values of 0.06 and 0.30, we categorized patients into three distinct risk groups: low-risk (DLCRs ≥0.30), intermediate-risk (DLCRs between 0.06 and 0.30), and high-risk (DLCRs ≤0.06). Significant differences in DFS were observed across patients in DLCR-defined low-risk, intermediate-risk, and high-risk groups in both training (Fig. 2A), internal testing (Fig. 2B), and external testing (Fig. 2C) sets (Log-rank test: all *P-*values<0.0001). Furthermore, as illustrated in Supplementary Table S2 (Supplemental Digital Content 2, http://links.lww.com/JS9/C772), the likelihood of tumor recurrence was notably higher in patients with higher DLCR risks. Specifically, compared to patients with low-risk, high-risk patients [hazard ratio (HR): 6.023, 95% CI: 2.490–14.570, *P*<0.0001] and intermediate-risk patients (HR: 2.836, 95% CI: 1.064–7.560; *P*=0.037) were, respectively, 6.023 and 2.836 times more likely to experience tumor recurrence in the external testing set (Supplementary Table S3, Supplemental Digital Content 2, http://links.lww.com/JS9/C772).

### Comparison of DLCR, Rad-score, and clinical model

Five radiomics features were identified as optimal for DFS prediction, and a radiomics model was constructed using the Multivariate Cox proportional hazards regression (Supplementary Fig. S2, Supplemental Digital Content 2, http://links.lww.com/JS9/C772). Rad-Score of each patient was calculated according to the features’ coefficients (Supplementary S5, Supplemental Digital Content 2, http://links.lww.com/JS9/C772), which allowed us to classify patients into low-risk, intermediate-risk, and high-risk groups. Supplementary Fig. S3 (Supplemental Digital Content 2, http://links.lww.com/JS9/C772) and Supplementary Table S2 (Supplemental Digital Content 2, http://links.lww.com/JS9/C772) indicated the significant DFS differences among these risk groups in the training, internal testing, and external testing sets (Log-rank test: all *P-*values<0.0001). Moreover, clinic-pathological characteristics were entered into the Univariate and Multivariate Cox proportional hazards regression, resulting in a clinical model consisting of Fuhrman grade, tumor size, and ECOG-PS (Supplementary Table S4, Supplemental Digital Content 2, http://links.lww.com/JS9/C772).

**Figure 3:**
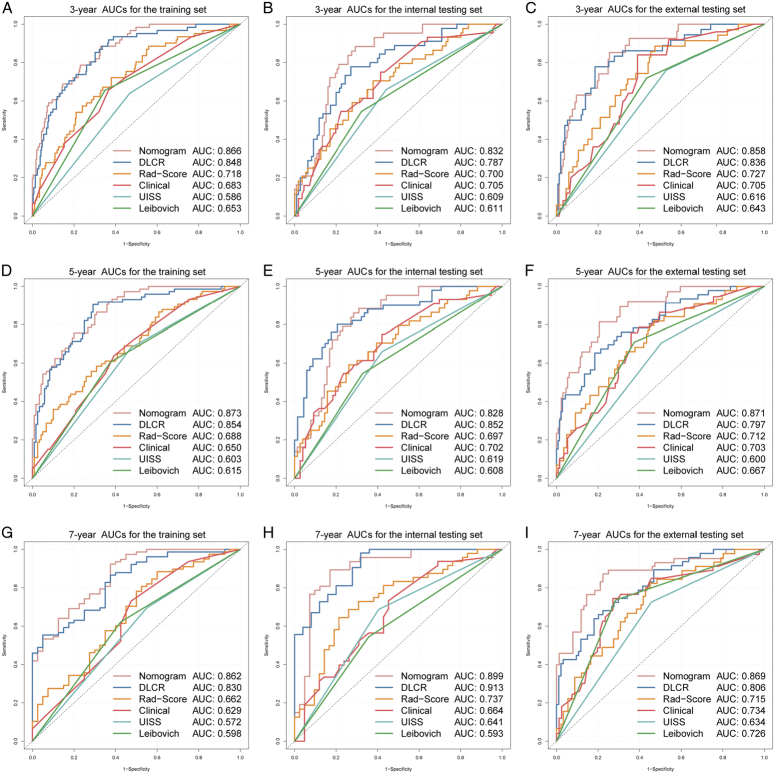
The time-dependent receiver operating characteristic (ROC) curves of the combined nomogram, deep learning computed risk score (DLCR), Rad-Score, Clinical model, UISS, and Leibovich score for disease-free survival (DFS) predictions of patients with localized ccRCC. 3-year ROC curves in the training (A), internal testing (B), and external testing (C) sets; 5-year ROC curves in the training (D), internal testing (E), and external testing (F) sets; 7-year ROC curves in the training (G), internal testing (H), and external testing (I) sets.

The DLCR outperformed both the Rad-Score and the clinical model in DFS prediction, exhibiting C-indexes of 0.804 vs. 0.703 vs. 0.663 in the training set, 0.781 vs. 0.686 vs. 0.683 in the internal test set, and 0.754 vs. 0.689 vs. 0.665 in the external testing set, respectively (Table 2). Figure 3 showcases the time-dependent ROC curves for the DLRC, Rad-Score, and clinical model. Within the external testing set, the DLCR achieved impressive time-dependent area under the curve (AUC) values of 0.836 (95% CI: 0.762–0.910), 0.797 (95% CI: 0.723–0.871), and 0.806 (95% CI: 0.732–0.881) for predicting DFS at 3, 5, and 7 years, respectively (Fig. 3 C, F, I, and Table 2). In comparison, the Rad-Score exhibited AUCs of 0.727 (95% CI: 0.639–0.815), 0.712 (95% CI: 0.627–0.796), and 0.715 (95% CI: 0.625–0.804), while the clinical model showed AUCs of 0.705 (95% CI: 0.611–0.800), 0.703 (95% CI: 0.614–0.792), and 0.734 (95% CI: 0.642–0.827).

### DLCR outperformed UISS and Leibovich models

The robustness of two traditional prognostic models, UISS and Leibovich, was further investigated and externally validated. While significant DFS differences were observed between the risk groups stratified by these models (Supplementary Fig S4, Supplemental Digital Content 2, http://links.lww.com/JS9/C772 and Supplementary S5, Supplemental Digital Content 2, http://links.lww.com/JS9/C772, Supplementary Table S2, Supplemental Digital Content 2, http://links.lww.com/JS9/C772), their predictive capabilities were found to be less effective compared to DLCR. In the external testing set, the C-indexes for UISS and Leibovich were 0.623 (95% CI: 0.553–0.694) and 0.663 (95% CI: 0.539–0.786), respectively. The DLCR demonstrated a more reliable DFS predicting performance in patients with localized ccRCC, evidenced by higher C-indexes and time-dependent AUC values across all sets (Table 2 and Fig. 3).

### Stratified analysis of DLCR in subgroups defined by clinic-pathological characteristics

**Figure 4:**
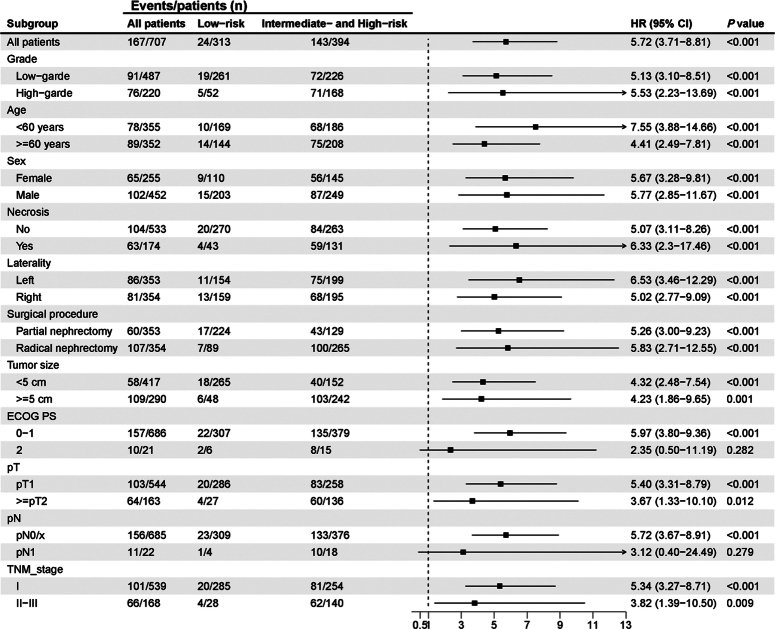
Stratified analysis of the deep learning computed risk score (DLCR) in subgroups defined by clinic-pathological characteristics.

Subgroup analysis was conducted across the entire study cohort to further assess the prognostic strength of the DLCR. The DLCR maintained its robust prognostic significance across subgroups classified by pathological grade, age, sex, pathological necrosis, tumor laterality, surgical procedure, tumor size, pT stage, and TNM stage (Fig. 4). No significant differences in DFS were observed in the ECOG-PS 1 (*P*=0.282) and pN 1 status (*P*=0.279) subgroups, which included relatively small sample sizes (*n*=21 and 22, respectively).

### Complementary value of DLCR to UISS, Leibovich score, and Rad-score

In the Kaplan–Meier survival analysis, DLCR-stratified patients showed significant DFS differences within UISS-defined low-risk (Supplementary Fig. S6A, Supplemental Digital Content 2, http://links.lww.com/JS9/C772) and intermediate-risk (Supplementary Fig. S6B, Supplemental Digital Content 2, http://links.lww.com/JS9/C772), as well as Leibovich-defined (Supplementary Fig. S7A-C, Supplemental Digital Content 2, http://links.lww.com/JS9/C772) risk subgroups (Log-rank test: all *P*-values <0.01). Additionally, patients were also precisely risk stratified using DLCR in the Rad-Score defined low-risk (Supplementary Fig. S8A, Supplemental Digital Content 2, http://links.lww.com/JS9/C772), intermediate-risk (Supplementary Fig. S8B, Supplemental Digital Content 2, http://links.lww.com/JS9/C772), and high-risk (Supplementary Fig. S8C, Supplemental Digital Content 2, http://links.lww.com/JS9/C772) subgroups (Log-rank test: all *P*-values<0.0001). Furthermore, in almost all subgroups delineated by UISS, Leibovich, and Rad-Score, patients identified as intermediate-risk or high-risk by DLCR had a higher likelihood of ccRCC recurrence compared to those in the low-risk category, as shown in Table 3.

**Table 3:** The hazard ratio (HR) estimates associated with DFS of different DLCR-defined risk groups in subgroups stratified by Rad-Score, UISS, and Leibovich.

Subgroup	Inter-group comparison	HR (95% CI)	*P*
Rad-Score low-risk	DLCR intermediate-risk VS low-risk	2.548 (1.170–5.547)	0.019*
Rad-Score low-risk	DLCR high-risk VS low-risk	4.964 (2.541–9.698)	<0.0001*
Rad-Score intermediate-risk	DLCR intermediate-risk VS low-risk	3.553 (1.350–9.349)	0.010*
Rad-Score intermediate-risk	DLCR high-risk VS low-risk	7.606 (3.184–18.169)	<0.0001*
Rad-Score high-risk	DLCR intermediate-risk VS low-risk	4.804 (1.862–12.390)	0.001*
Rad-Score high-risk	DLCR high-risk VS low-risk	12.309 (5.425–27.930)	<0.0001*
UISS low-risk	DLCR intermediate-risk VS low-risk	3.358 (1.451–7.773)	0.005*
UISS low-risk	DLCR high-risk VS low-risk	8.779 (4.213–18.294)	<0.0001*
UISS intermediate-risk	DLCR intermediate-risk VS low-risk	3.105 (1.552–6.308)	0.001*
UISS intermediate-risk	DLCR high-risk VS low-risk	5.486 (2.968–10.141)	<0.0001*
UISS high-risk	DLCR intermediate-risk VS low-risk	1.756 (0.293–10.530)	0.538
UISS high-risk	DLCR high-risk VS low-risk	3.984 (0.879–18.060)	0.073
Leibovich low-risk	DLCR intermediate-risk VS low-risk	3.086 (1.590–5.989)	0.001*
Leibovich low-risk	DLCR high-risk VS low-risk	6.219 (3.374–11.466)	<0.0001*
Leibovich intermediate-risk	DLCR intermediate-risk VS low-risk	2.198 (0.805–6.001)	0.124
Leibovich intermediate-risk	DLCR high-risk VS low-risk	4.102 (1.636–10.284)	0.003*
Leibovich high-risk	DLCR intermediate-risk VS low-risk	6.972 (0.829–57.950)	0.072
Leibovich high-risk	DLCR high-risk VS low-risk	14.393 (1.957–105.870)	0.009*

Note: *P-*value was calculated via Univariate Cox analysis.

* *P-*value＜0.05;

CI, confidential interval.

**Figure 5:**
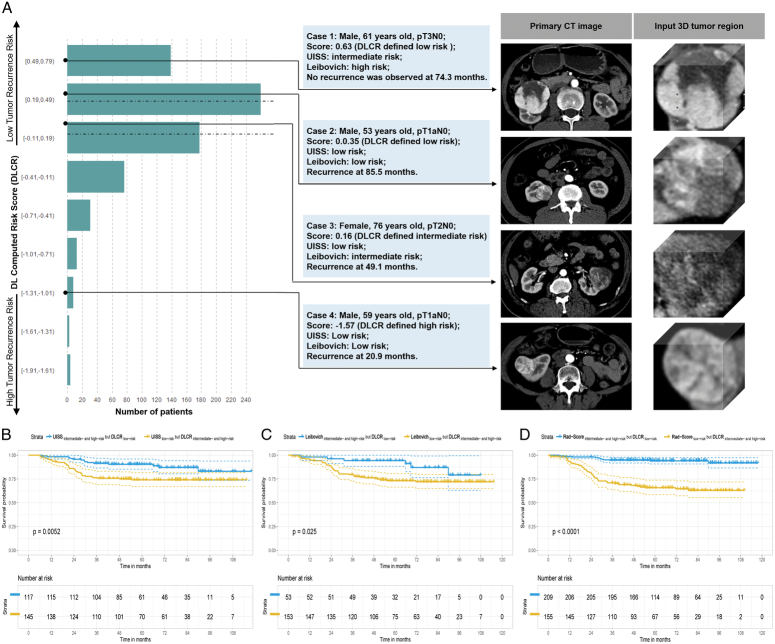
Distribution of deep learning computed risk scores (DLCRs), and four representative patients with inconsistent risks as defined by DLCR, UISS, and Leibovich (A). Kaplan–Meier survival analysis for disease-free survival (DFS) comparing patients in UISS (B), Leibovich (C), and Rad-Score (D) determined intermediate-risk and high-risk but DLCR-defined low-risk groups with those of low-risk but DLCR-defined intermediate-risk and high-risk groups.

The distribution of DLCRs in the whole study cohort, and four representative patients with inconsistent risks as defined by DLCR, UISS, and Leibovich are shown in Figure 5A. To be noted, significantly shorter DFS was observed in the subgroup of patients classified as low-risk by UISS but intermediate-risk and high-risk by DLCR compared to those categorized as intermediate-risk and high-risk by UISS but low-risk by DLCR (HR: 2.344, 95% CI: 1.267–4.337, Log-rank test: *P*=0.0052; Fig. 5B). Furthermore, as depicted in Figure 5C, patients stratified as low-risk by the Leibovich score but intermediate-risk and high-risk by DLCR had a 2.578-fold higher likelihood of experiencing ccRCC recurrences than those classified as intermediate-risk and high-risk by Leibovich but low-risk by DLCR (HR: 2.578, 95% CI: 1.094–6.073, Log-rank test: *P*=0.025). Additionally, the Kaplan–Meier survival analysis revealed a similar trend in the comparison of Rad-Score stratified low-risk patients who were deemed intermediate-risk and high-risk by DLCR against those labeled as intermediate-risk and high-risk by Rad-Score but low-risk by DLCR, indicating a significantly higher risk (HR: 6.679, 95% CI: 3.643–12.240, Log-rank test: *P*<0.0001; Fig. 5D).

### Construction of a user-friendly combined nomogram

**Figure 6:**
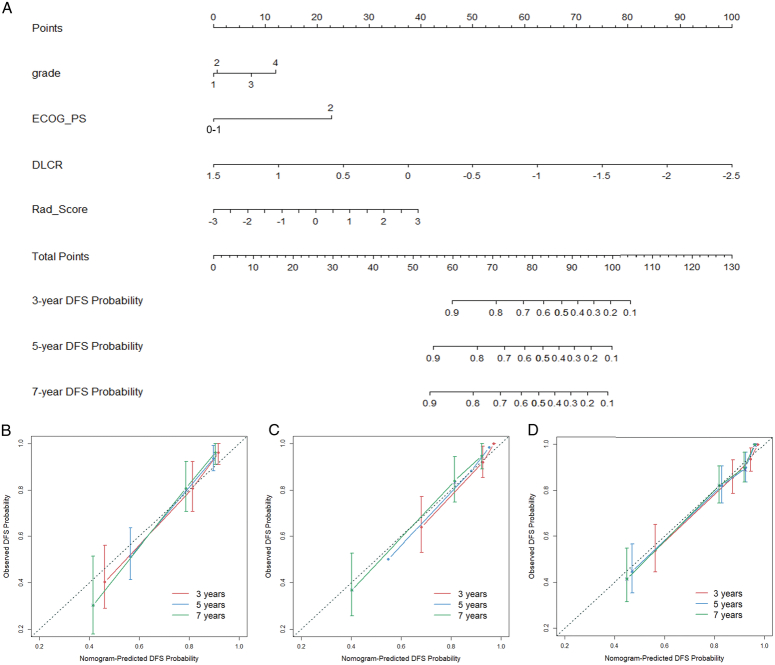
A combined nomogram (A) integrating ECOG-PS, Fuhrman grade, Rad-Score, and DLCR to predict DFS in patients with localized ccRCC. The calibration curves revealed good agreements between nomogram-predicted and observed disease-free survival (DFS) probabilities for 3-year, 5-year, and 7-year DFS prediction, in the training (B), internal testing (C), and external testing (D) sets.

As illustrated in Supplementary Fig. S9 (Supplemental Digital Content 2, http://links.lww.com/JS9/C772), after adjusting for clinic-pathological prognostic variables and Rad-Score, the DLCR continued to demonstrate significant differences in DFS for patients with localized ccRCC in the training set (*P*<0.001). A combined nomogram was thereby constructed, which consisted of Fuhrman grade, ECOG-PS, Rad-Score, and DLCR (Fig. 6A). The nomogram presented enhanced predicting ability, achieving C-indexes of 0.803 (95% CI: 0.750–0.855) and time-dependent AUCs of 0.858 (95% CI: 0.789–0.927), 0.871 (95% CI: 0.811–0.931), and 0.869 (95% CI: 0.801–0.937) for 3, 5, and 7 years, respectively, in the external validation set (Table 2 and Fig. 3). The Calibration curve analysis demonstrated good concordance between nomogram-predicted and observed DFS probabilities in the training (Fig. 6B), internal testing (Fig. 6C), and external testing (Fig. 6D) sets.

## Discussion

Up to 30% of patients are at risk of tumor recurrence after nephrectomy, increasing the need to explore novel preoperative prognostic factors that enable accurate DFS prediction in localized ccRCC to facilitate risk stratification and clinical decision-making^35^. In this retrospective multicentre study, we developed and externally validated a DL prognostic model for DFS prediction in patients with localized ccRCC based on preoperative CT scans. The constructed DLCR can perfectly stratify patients into distinct risk groups, outperforming the Rad-Score and clinical model we built, as well as two existing prognostic models – UISS and Leibovich score. Most notably, the DLCR provided complementary values to UISS, Leibovich, and Rad-Score via a more accurate DFS risk stratification of localized ccRCC patients. Furthermore, the DLCR proved to be a novel prognostic factor that independent of clinical features.

Few previous studies have investigated the prognostic value of CT-based DL models in patients with ccRCC, revealing the capability of DL in analyzing tumor heterogeneity prognostic information derived from medical images and obtaining C-indexes around 0.710 in the testing sets^36–38^. In consistence with them, using preoperative CMP CT images, we successfully trained a DL prognostic model and calculated DLCRs of patients with localized ccRCC, which enabled perfect patients’ DFS risk stratification. The DLCR we built achieved more favorable predictive performances, proved by C-indexes of 0.781 (95% CI: 0.728–0.833) and 0.754 (95% CI: 0.689–0.821) in the internal and external testing sets, respectively. Besides, superior to most previous investigations that utilizes single-center, small-sample data to architect their models, a total of 707 patients with localized ccRCC from six independent medical centers were enrolled in our study, which brought a better generalizability. It is worth stating that although the inclusion time period of the training and internal testing sets differed from the external testing set, there were no significant differences between the three data sets for almost all baseline characteristics, demonstrating a minimal selection bias. Furthermore, enrolling patients during a different time period as an external test can better simulate real-world scenarios. Our DL model yielded satisfactory predictive performance in the external testing set, with time-dependent AUC values of 0.836 (95% CI: 0.762–0.910), 0.797 (95% CI: 0.723–0.871), and 0.806 (95% CI: 0.732–0.881) for predicting DFS at 3, 5, and 7 years, respectively. These results suggest that our model has robust generalization capabilities across diverse data sources and scenario, which is in line with studies of similar patient cohorts^39,40^.

More previous studies have demonstrated the predictive abilities of the CT-based radiomics models for ccRCC patients’ outcomes, while few of them compared their established models with the existing traditional models (such as UISS and Leibovich score)^41–43^. Besides, the superiority in prognosis prediction of ccRCC patients between radiomics and DL models remains unexplored. Therefore, this study constructed a radiomics model (Rad-Score) as well, and compared it with DLCR, clinical model, UISS, and Leibovich score. As a result, the DLCR (C-indexes: 0.781 [95% CI: 0.728–0.833] and 0.754 [95% CI: 0.689–0.821] in the internal and external testing sets, retrospectively) presented the most outstanding DFS predicting capability in localized ccRCC patients, followed by the Rad-Score (internal: 0.686 [95% CI: 0.614–0.758]; external: 0.689 [95% CI: 0.617–0.760]) and clinical model (internal: 0.683 [95% CI: 0.603–0.763]; external: 0.665 [95% CI: 0.597–0.734]), which were both superior to the UISS (internal: 0.611 [95% CI: 0.531–0.691]; external: 0.623 [95% CI: 0.553–0.694]) and Leibovich (internal: 0.609 [95% CI: 0.535–0.683]; external: 0.663 [95% CI: 0.539–0.786]) models. Differed from radiomics method that relies on multiple manually dependent sequential steps such as tumor segmentation, feature extraction and selection, the extraordinary DFS predicting the performance of DLCR may originate from the end-to-end nature of the DL model that eliminates instabilities of multistep manual involvements.

In a more recent study, Yang *et al*.^24^ employed radiomics features to develop a radiomics signature for recurrence-free survival prediction in patients with localized ccRCC, comparing their radiomics signature with UISS and Leibovich models and exploring the incremental value of radiomics to existing traditional models. The predictive performances of UISS and Leibovich models in their study were comparable with ours, and they drew the same conclusion regarding the better prognostic value of radiomics models. Differed from them, we employed a more robust DL model, and further explored the risk stratification ability of our DLCR in subgroups determined by clinic-pathological features, UISS, Leibovich score, and Rad-Score. The DLCR facilitated DFS risk stratifications in almost all subgroups, except for those with limited patient numbers. Furthermore, a critical innovation of our study is the DLCR’s complementary role alongside traditional models like UISS and Leibovich, as well as the Rad-Score. This was particularly evident in subgroups where patients stratified as low-risk by UISS/Leibovich/Rad-Score, yet identified as intermediate- or high-risk by DLCR, exhibited a significantly higher likelihood of ccRCC recurrence. Verifying a more sophisticated DFS risk differentiation ability of the DLCR, which is meaningful in more informed clinical decisions. Moreover, the DLCR remained a strong prognostic factor after adjusting Rad-Score and clinic-pathological risk factors, and a clinically useful combined nomogram with increased predictive performance and fine agreements between predicted-DFS and observed-DFS probabilities was formed. The proposed DLCR acted as a novel prognostic factor in patients with localized ccRCC and the CT-based 3D DL model may enlighten prognosis biomarkers’ exploration in advanced ccRCC.

Several limitations of this study should be noted. Firstly, as a multicentre retrospective study, the selection bias is inevitable. Moreover, although the model was trained and validated on patients from six independent centers, a larger scale of international cohorts is still needed. In addition, manually segmenting tumor area is not only time-consuming and labor-intensive, but also highly subjective due to the inconsistences cross the readers, a fully automated precision sketching tool is therefore required. Fourthly, utilizing whole-slide histopathological images (WSI) and genomic information alongside CT images as inputs may enhance the predictive performance of DL models. Our future research will explore the use of multiomics data in DL models for postoperatively predicting DFS in ccRCC. Last but not least, the current study enrolled localized ccRCC patients only. In the future, we will explore the predictive values of medical image-based DL models in overall survival and effectiveness of adjuvant therapies for advanced ccRCC.

## Conclusion

In conclusion, the DL computed score based on CT scan enables accurate preoperative prognostic prediction of patients with localized ccRCC and is superior to the constructed radiomics, clinical models and existing UISS, Leibovich models. The DLCR can be used to complement traditional models and Rad-Score by providing a more precise way of risk stratifying patients, which may benefit more informed treatment decisions and adoption of adjuvant therapies.

## Ethical approval

The ethic approval was obtained from the institutional review board of the First Affiliated Hospital of Chongqing Medical University (2022-KY508).

## Consent

The patients’ informed consent was waived due to the retrospective nature of our study.

## Source of funding

This study was founded by the Chongqing Talent Program (grant number: 202003), the National Key R&D Program of China (grant number: 2020YFA0714002), and the Chongqing Municipal Education Co1runission's 14th Five-Year Key Discipline Support Project (grant number: 20240101).

## Author contribution

Y.X., Z.W., and M.X.: conception and design; Y.X.: administrative support; Y.X., Z.W., S.Y., and H.T.: data collection; Y.X. and Z.W.: data analysis and interpretation; Y.X.: deep learning methods; Y.X., Z.W., and Y.L.: manuscript writing. All authors contributed to the manuscript review and approved the submitted version.

## Conflicts of interest disclosure

The authors declare no conflicts of interest in this study.

## Research registration unique identifying number (UIN)

1. Name of the registry: DL-ccRCC.
2. Unique identifying number or registration ID: NCT06088134.
3. Hyperlink to your specific registration (must be publicly accessible and will be checked): https://clinicaltrials.gov/study/NCT06088134.

## Guarantor

Mingzhao Xiao.

## Data availability statement

The original data are available from the corresponding author-Mingzhao Xiao upon reasonable request. The source code for the deep learning model is available online (https://github.com/wzj9846/ccRCC_prognosis).

## Provenance and peer review

Not commissioned, externally peer-reviewed.

## Supplementary Material
