# Supplementary material for: Three-dimensional deep learning model complements existing models for preoperative disease-free survival prediction in localized clear cell renal cell carcinoma: a multicenter retrospective cohort study
Source: Int J Surg. 2024 Jun 19;110(11):7034–46. doi: 10.1097/JS9.0000000000001808 (PMC11573058; doi:10.1097/JS9.0000000000001808)
Supplement: Supplementary file 2 [file js9-110-7034-s002.docx]

**Supplementary content to:** **Three-Dimensional (3D) Deep Learning Model Complements Existing Models for Preoperative Disease-Free Survival Prediction (DFS) in Localized Clear Cell Renal Cell Carcinoma (ccRCC): A Multicenter Retrospective Cohort Study**

***Supplementary Materials Catalogue***

***Supplementary Figures***

1. ***Fig. S1*** *The overall patients’ recruitment and data split procession*
2. ***Fig. S2*** *The selection of prognostic radiomics features*
3. ***Fig. S3*** *T The Kaplan-Meier survival analysis of patients with different risks in the training (A), internal testing (B) and external testing (C) sets stratified by Rad-Score*
4. ***Fig. S4*** *The Kaplan-Meier survival analysis of patients with different risks in the training (A), internal testing (B) and external testing (C) sets stratified by UISS model*
5. ***Fig. S5*** *The Kaplan-Meier survival analysis of patients with different risks in the training (A), internal testing (B) and external testing (C) sets stratified by Leibovich score*
6. ***Fig. S6*** *Kaplan-Meier survival analysis of patients with low-, intermediate-, and high-DLCR risk in the UISS defined low- (A), intermediate- (B), and high-risk (C) subgroups*
7. ***Fig. S7*** *Kaplan-Meier survival analysis of patients with low-, intermediate-, and high-DLCR risk in the 2003 Leibovich defined low- (A), intermediate- (B), and high-risk (C) subgroups.*
8. ***Fig. S8*** *Kaplan-Meier survival analysis of patients with low-, intermediate-, and high-DLCR risk in the Rad-Score defined low- (A), intermediate- (B), and high-risk (C) subgroups.*
9. ***Fig. S9*** *The forest plot for Multivariate Cox analysis of clinic-pathological prognostic factors, Rad-Score, and DLCR*

***Supplementary Tables***

1. ***Table S1*** *The detailed parameters of CT scanners*
2. ***Table S2*** *C-indexes of 3D ResNet-50, 3D ResNet-18, and 3D VGG-16 in localized ccRCCs’ DFS prediction for training and internal testing sets*
3. ***Table S3*** The hazard ratio (HR) estimates associated with DFS in different risk groups and subgroups stratified by DLCR, Rad-Score, UISS and Leibovich
4. ***Table S4*** *Univariate and multivariate Cox proportional hazards regression analysis of clinic-pathological features*

***Other Supplementary***

1. ***Supplementary S1.*** *The evaluations of UISS stage and Leibovich score*
2. ***Supplementary S2.*** *Definitions of various CT phases*
3. ***Supplementary S3.*** *The specific classifications of extracted radiomics features*
4. ***Supplementary S4.*** *Radiomics feature demission reduction procession*
5. ***Supplementary S5.*** *The calculation formula of the Rad-Score*
6. ***Fig. S1 The overall patients’ recruitment and data split procession***


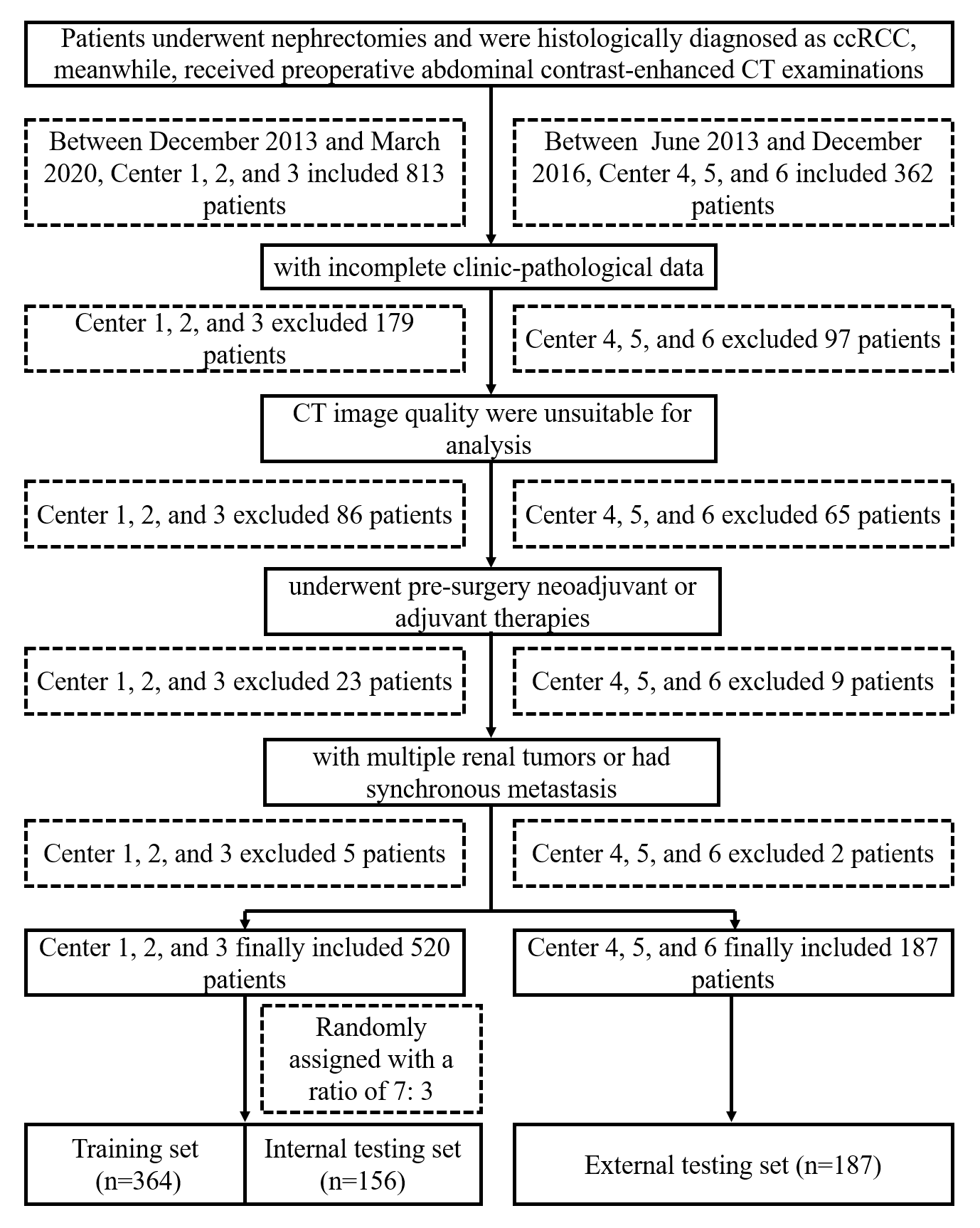


1. ***Fig. S2 The selection of prognostic radiomics features. (A) A Generated coefficient profile plot based on the selected values of logarithm λ; (B) Five radiomics features were finally chosen*** ***by the least absolute shrinkage and selection operator (LASSO) Cox algorithm at the in Tuning parameter (λ) of 0.0450474; (C) The weights plot of the selected features.***


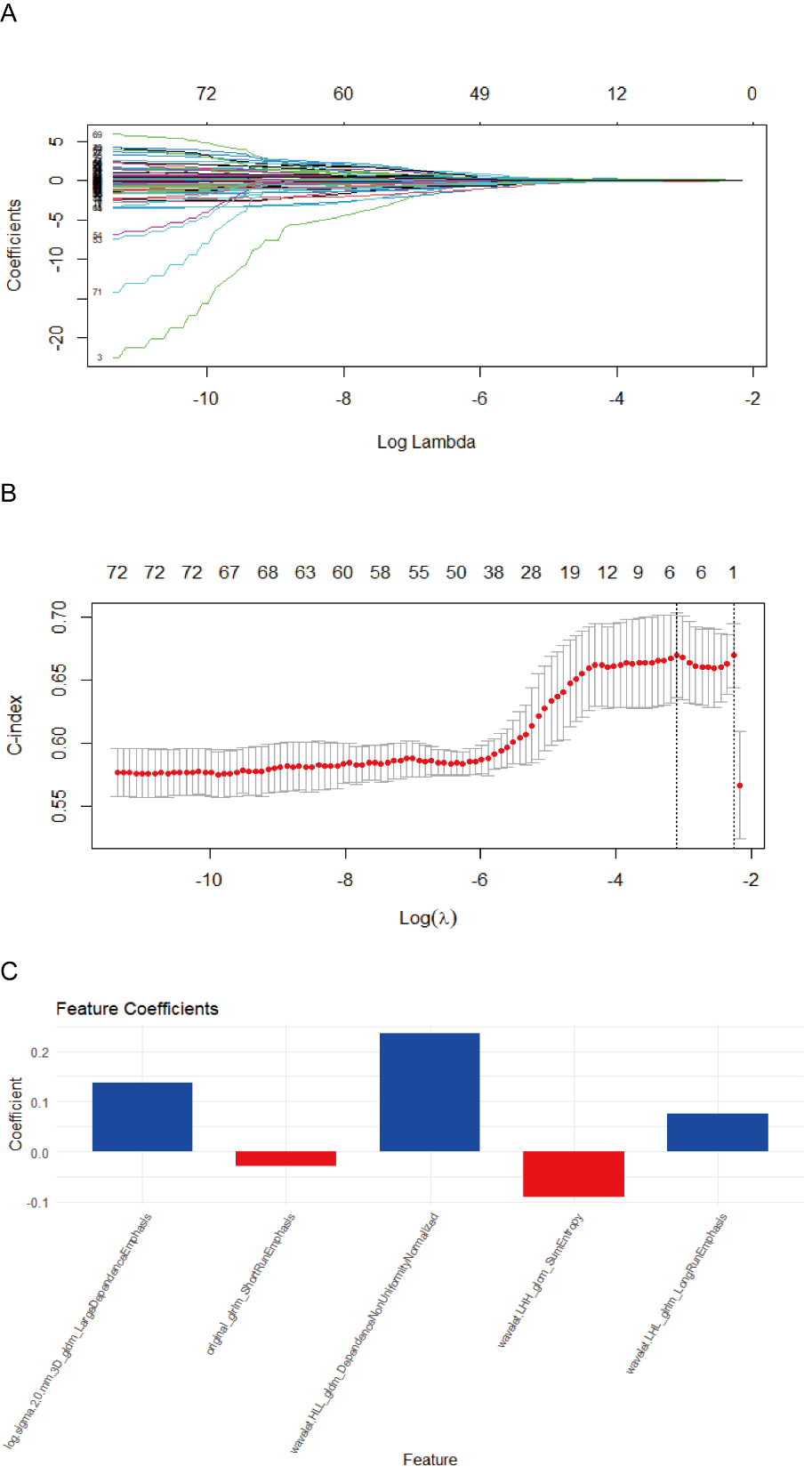


1. ***Fig. S3 T The Kaplan-Meier survival analysis of patients with different risks in the training (A), internal testing (B) and external testing (C) sets stratified by Rad-Score.***


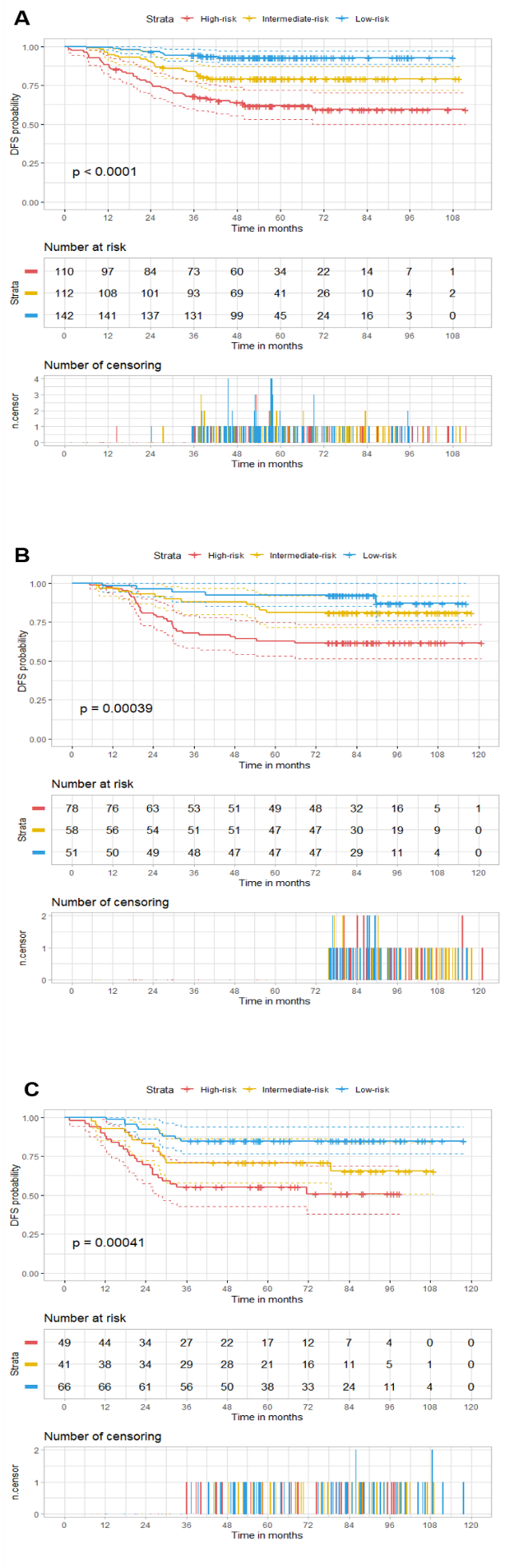


1. ***Fig. S4 The Kaplan-Meier survival analysis of patients with different risks in the training (A), internal testing (B) and external testing (C) sets stratified by UISS model.***


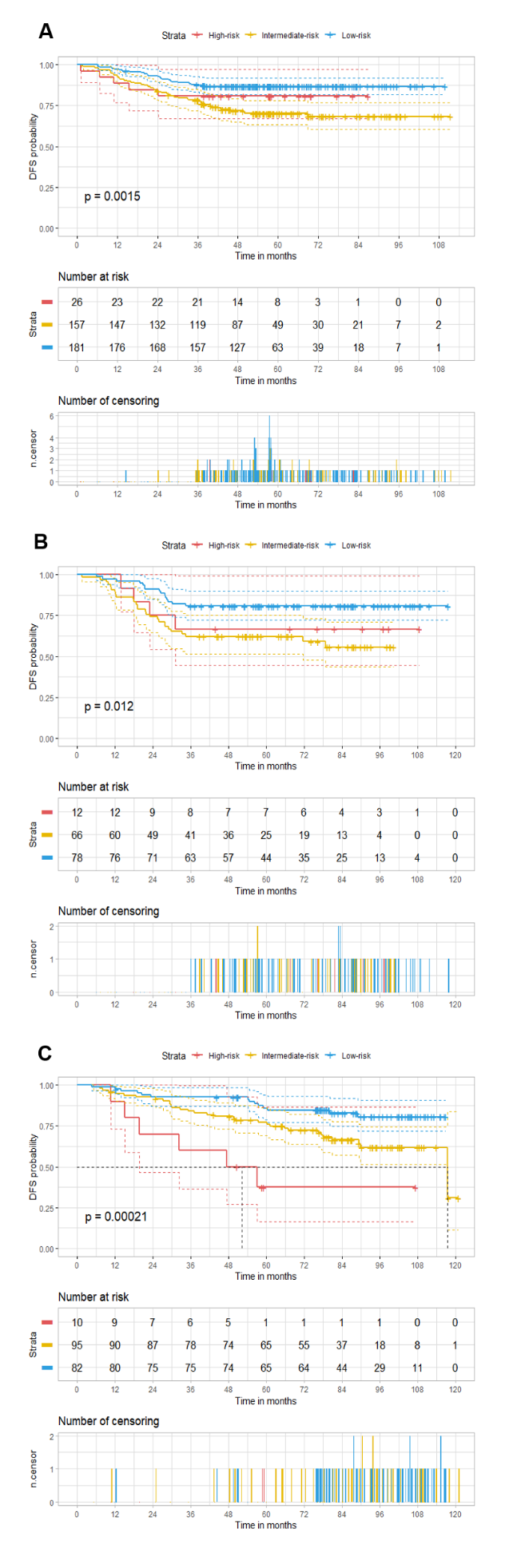


1. ***Fig. S5 The Kaplan-Meier survival analysis of patients with different risks in the training (A), internal testing (B) and external testing (C) sets stratified by Leibovich score.***


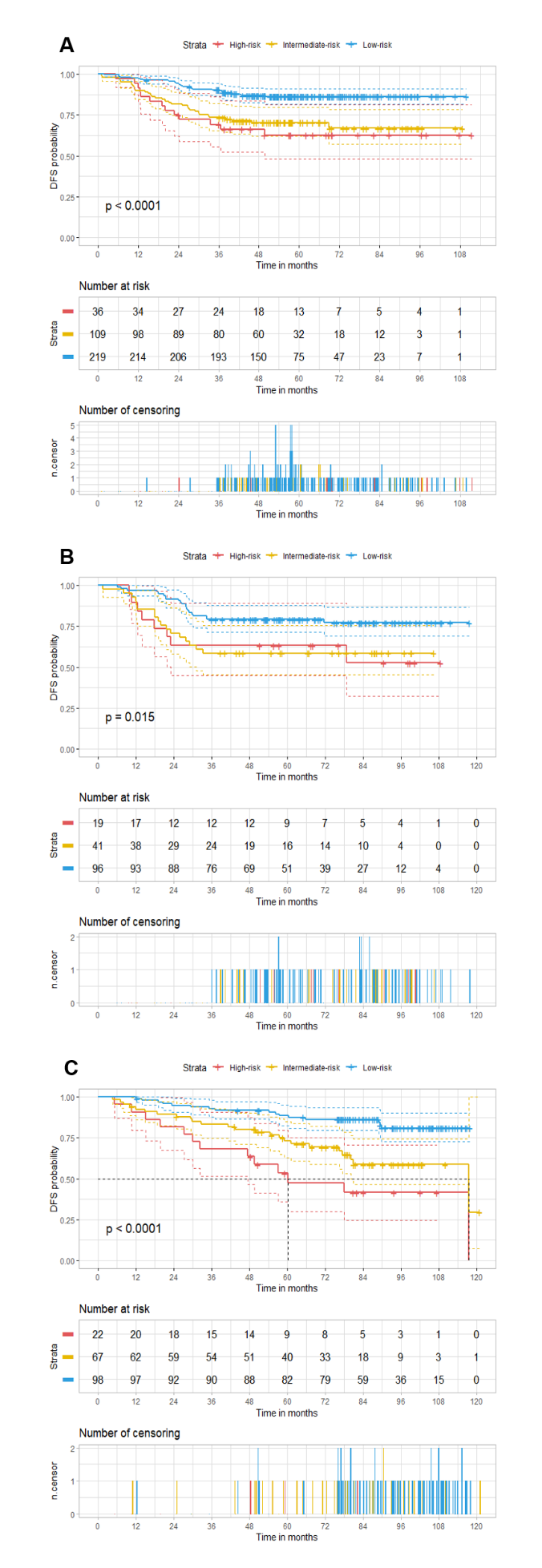


1. ***Fig. S6 Kaplan-Meier survival analysis of patients with low-, intermediate-, and high-DLCR risk in the UISS defined low- (A), intermediate- (B), and high-risk (C) subgroups.***


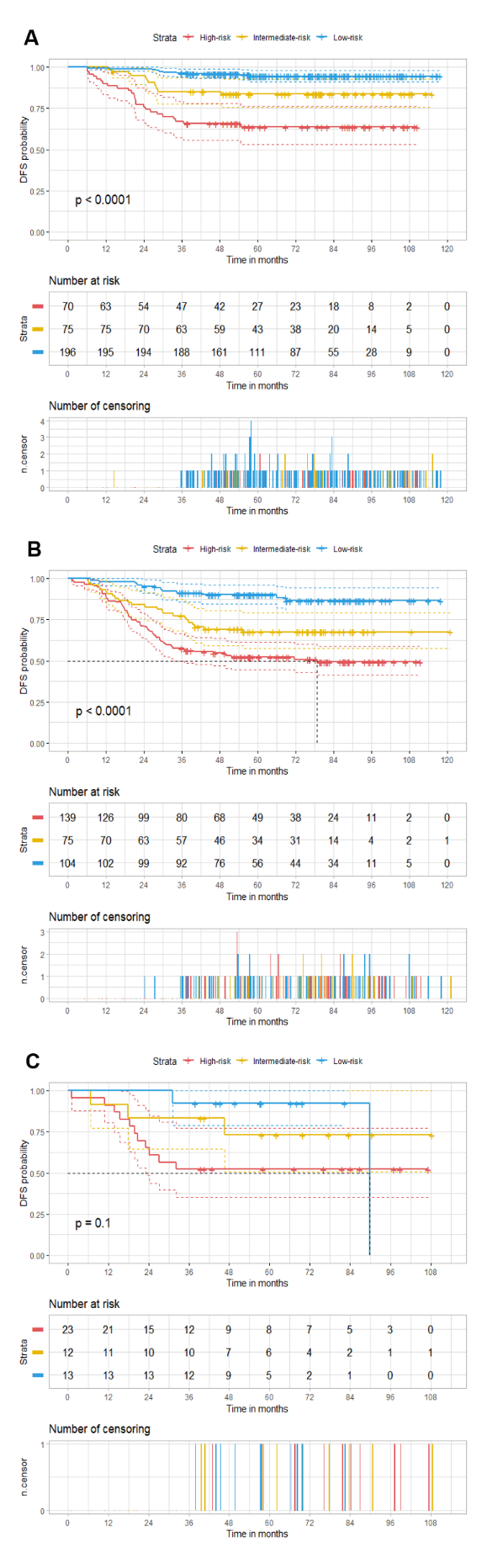


1. ***Fig. S7 Kaplan-Meier survival analysis of patients with low-, intermediate-, and high-DLCR risk in the Leibovich defined low- (A), intermediate- (B), and high-risk (C) subgroups.***


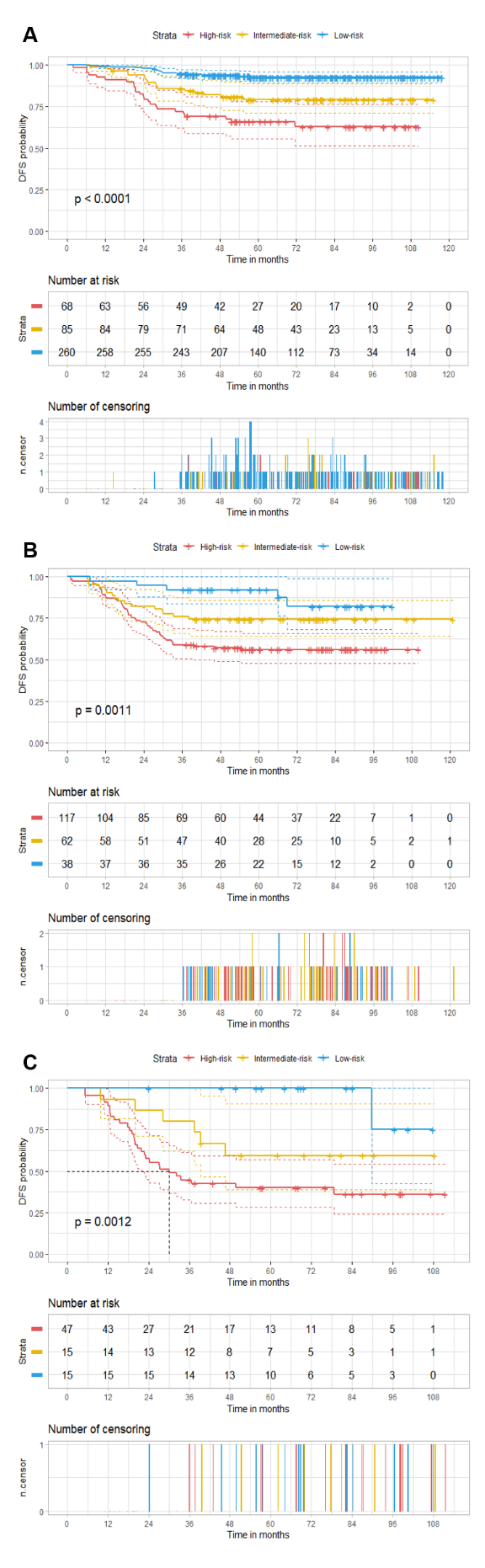


1. ***Fig. S8 Kaplan-Meier survival analysis of patients with low-, intermediate-, and high-DLCR risk in the Rad-Score defined low- (A), intermediate- (B), and high-risk (C) subgroups.***


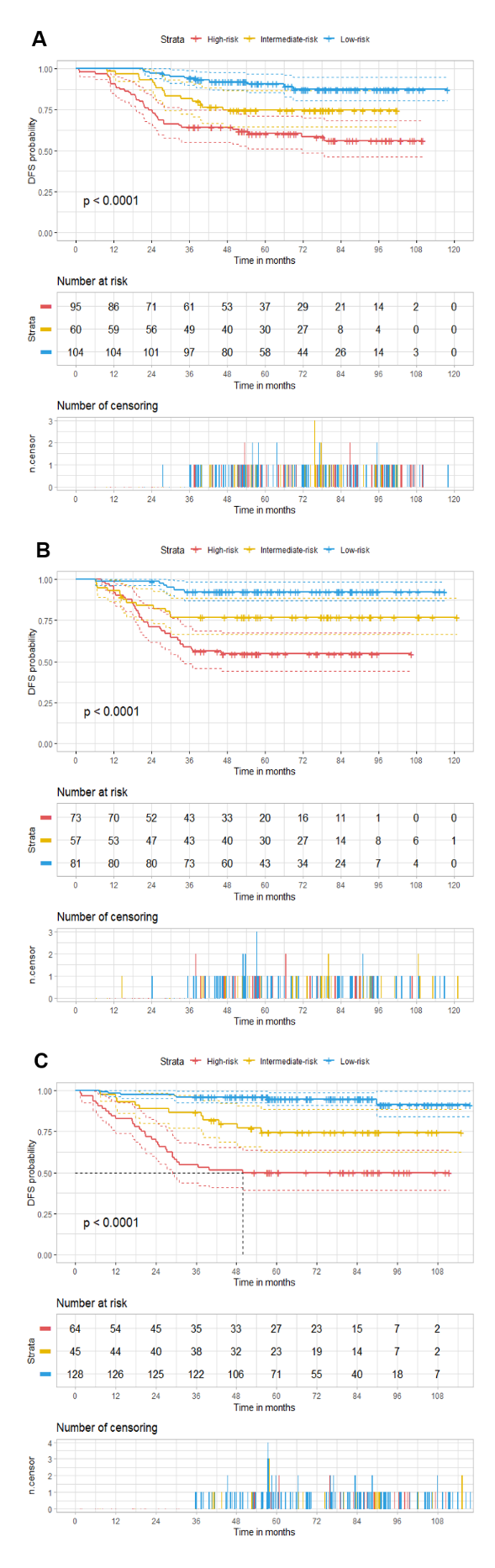


1. ***Fig. S9 The forest plot for Multivariate Cox analysis of clinic-pathological prognostic factors, Rad-Score, and DLCR. After adjusting for clinic-pathological prognostic variables and Rad-Score, the DL-score still showed significant differences in DFS for patients with localized ccRCC in the training set (p < 0.001) in the multivariate Cox regression analysis.***


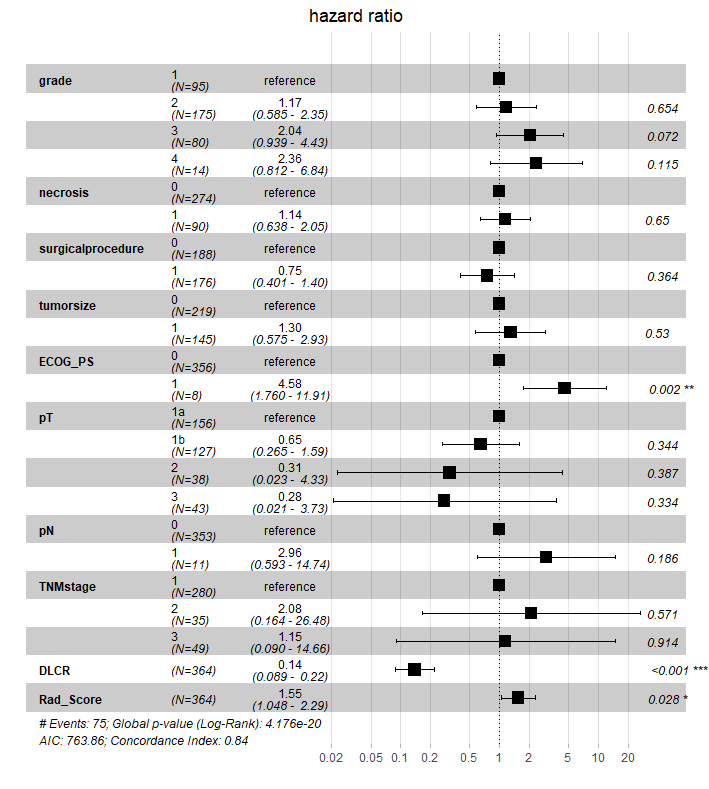


1. ***Table S1. The detailed parameters of CT scanners***

| **CT scanner** | Discovery CT750 HD (GE  Healthcare, USA) | LightSpeed VCT (GE  Healthcare, USA) | Somatom Definition Flash  (Siemens Healthcare,  Germany) | Somatom Definition (Siemens Healthcare, Germany) | Somatom Sensation 64 (Siemens Healthcare, Germany) | Brilliance iCT 256 (Philips, Netherlands) |
| --- | --- | --- | --- | --- | --- | --- |
| Tube voltage (kV) | 100-120 | 100-120 | 100-120 | 100-120 | 100-120 | 100-120 |
| Tube current (mA) | 200-400 | 150-300 | 200 | 200 | 200 | 250 |
| Gantry rotation time (s) | 0.5 | 0.5 | 0.28 | 0.33 | 0.5 | 0.5 |
| Detector collimation (mm) | 0.625 | 0.625 | 0.6 | 0.6 | 0.6 | 0.75 |
| Image matrix | 512*512 | 512*512 | 512*512 | 512*512 | 512*512 | 512*512 |
| Slice thickness (mm) | 1-7 | 3-7 | 1-5 | 1-5 | 1-7 | 1-7 |
| Hospitals | Center 1, 2 | Center 1, 4, 6 | Center 2, 3, 5 | Center 3, 6 | Center 2, 5 | Center 1, 2, 4 |

Note: kV (kilovolt); mA (milliampere); s (second); mm (milimetre);

1. **Table S2** C-indexes of 3D ResNet-50, 3D ResNet-18, and 3D ResNet-101 in localized ccRCCs’ DFS prediction for training and internal testing sets

| Network architecture | Training set | Internal testing set |
| --- | --- | --- |
| 3D ResNet-50 | 0.804 | 0.781 |
| 3D ResNet-18 | 0.752 | 0.701 |
| 3D ResNet-101 | 0.726 | 0.695 |

1. **Table S3** The hazard ratio (HR) estimates associated with DFS in different risk groups and subgroups stratified by DLCR, Rad-Score, UISS and Leibovich

| Patients | Group/subgroup | HR | 95% CI | *P* value^1^ |
| --- | --- | --- | --- | --- |
| Training set | DLCR intermediate-risk VS low-risk | 4.826 | 2.228-10.460 | <0.0001* |
| Training set | DLCR high-risk VS low-risk | 10.037 | 5.067-19.880 | <0.0001* |
| Training set | Rad-Score intermediate-risk VS low-risk | 3.108 | 1.479-6.530 | 0.003* |
| Training set | Rad-Score high-risk VS low-risk | 6.605 | 3.313-13.170 | <0.0001* |
| Training set | UISS intermediate-risk VS low-risk | 2.414 | 1.474-3.956 | 0.0004* |
| Training set | UISS high-risk VS low-risk | 1.603 | 0.611-4.201 | 0.337 |
| Training set | Leibovich intermediate-risk VS low-risk | 2.574 | 1.562-4.239 | 0.0002* |
| Training set | Leibovich high-risk VS low-risk | 3.252 | 1.689-6.259 | 0.0004* |
| Internal testing set | DLCR intermediate-risk VS low-risk | 2.416 | 0.932-6.264 | 0.070 |
| Internal testing set | DLCR high-risk VS low-risk | 6.078 | 2.772-13.330 | <0.0001* |
| Internal testing set | Rad-Score intermediate-risk VS low-risk | 2.340 | 1.026-5.338 | 0.043* |
| Internal testing set | Rad-Score high-risk VS low-risk | 4.050 | 1.925-8.521 | 0.0002* |
| Internal testing set | UISS intermediate-risk VS low-risk | 2.517 | 1.338-4.735 | 0.004* |
| Internal testing set | UISS high-risk VS low-risk | 1.879 | 0.623-5.662 | 0.263 |
| Internal testing set | Leibovich intermediate-risk VS low-risk | 2.271 | 1.198-4.307 | 0.012* |
| Internal testing set | Leibovich high-risk VS low-risk | 2.362 | 1.046-5.337 | 0.039* |
| External testing set | DLCR intermediate-risk VS low-risk | 2.836 | 1.064-7.560 | 0.037* |
| External testing set | DLCR high-risk VS low-risk | 6.023 | 2.490-14.570 | <0.0001* |
| External testing set | Rad-Score intermediate-risk VS low-risk | 2.018 | 0.701-5.808 | 0.193 |
| External testing set | Rad-Score high-risk VS low-risk | 4.733 | 1.835-12.207 | 0.001* |
| External testing set | UISS intermediate-risk VS low-risk | 2.184 | 1.161-4.109 | 0.015* |
| External testing set | UISS high-risk VS low-risk | 6.359 | 2.412-16.768 | 0.0002* |
| External testing set | Leibovich intermediate-risk VS low-risk | 2.739 | 1.449-5.180 | 0.002* |
| External testing set | Leibovich high-risk VS low-risk | 5.083 | 2.426-10.650 | <0.0001* |

^1^ *P* value was calculated via Univariate Cox analysis; * *P* value＜0.05

1. ***Table S4 Univariate and multivariate Cox proportional hazards regression analysis of clinic-pathological features***

| Variable | Univariate Cox regression |  |  | Multivariate Cox regression |  |
| --- | --- | --- | --- | --- | --- |
|  | HR (95%CI) | *P* value |  | HR (95%CI) | *P* value |
| Age |  |  |  |  |  |
| ＜60 years | 1(ref) | - |  | - | - |
| ≥60 years | 1.518 (0.959-2.404) | 0.075 |  | - | - |
| Sex |  |  |  |  |  |
| Female | 1(ref) | - |  | - | - |
| Male | 0.842 (0.521-1.361) | 0.482 |  | - | - |
| Tumor laterality |  |  |  |  |  |
| Right | 1(ref) | - |  | - | - |
| Left | 0.859 (0.545-1.354) | 0.513 |  | - | - |
| Surgery procedure |  |  |  |  |  |
| Partial | 1(ref) | - |  | 1(ref) | - |
| Radical | 1.796 (1.128-2.859) | 0.014^*^ |  | 1.095 (0.584-2.055) | 0.776 |
| Tumor size |  |  |  |  |  |
| ＜5 cm | 1(ref) | - |  | 1(ref) | - |
| ≥5 cm | 2.414 (1.524-3.824) | <0.001^*^ |  | 2.079 (0.919-4.704) | 0.079 |
| Pathological necrosis |  |  |  |  |  |
| Absent | 1(ref) | - |  | 1(ref) | - |
| Present | 2.089 (1.312-3.326) | 0.002^*^ |  | 1.177 (0.658-2.106) | 0.583 |
| Fuhrman grade |  |  |  |  |  |
| I | 1(ref) | - |  | 1(ref) | - |
| II | 1.526 (0.788-2.955) | 0.210 |  | 1.307 (0.664-2.570) | 0.438 |
| Ⅲ | 2.575 (1.281-5.175) | 0.008^*^ |  | 1.924 (0.895-4.135) | 0.094 |
| Ⅳ | 4.874 (1.917-12.39) | 0.001^*^ |  | 3.201 (1.127-9.091) | 0.029^*^ |
| pT stage |  |  |  |  |  |
| T1a | 1(ref) | - |  | 1(ref) | - |
| T1b | 1.570 (0.905-2.726) | 0.109 |  | 0.762 (0.316-1.836) | 0.544 |
| T2 | 3.002 (1.543-5.839) | 0.001^*^ |  | 2.458 (0.175-34.477) | 0.504 |
| T3 | 1.736 (0.826-3.649) | 0.145 |  | 0.787 (0.083-7.432) | 0.835 |
| pN status |  |  |  |  |  |
| N0/Nx | 1(ref) | - |  | 1(ref) | - |
| N1 | 2.452 (0.989-6.077) | 0.053 |  | - | - |
| TNM stage |  |  |  |  |  |
| I | 1(ref) | - |  | 1(ref) | - |
| II | 2.23 (1.187-4.191) | 0.013^*^ |  | 0.375 (0.028-4.982) | 0.458 |
| Ⅲ | 1.611 (0.875-2.965) | 0.126 |  | 0.791 (0.100-6.259) | 0.824 |
| ECOG-PS |  |  |  |  |  |
| 0-1 | 1(ref) | - |  | 1(ref) | - |
| 2 | 4.366 (1.76-10.828) | 0.001^*^ |  | 5.562 (2.195-14.095) | <0.001^*^ |

* : *P*＜0.05

1. ***Supplementary S1. The evaluations of UISS stage and Leibovich score***

The **UISS stage** of each patient was evaluated based on specific criteria:

1. Patients with T1 stage, grade 1-2, and ECOG PS 0 are classified as low risk.
2. Patients with T3 stage, grade 2-4, and ECOG PS ≥1 or T4 stage are classified as high risk.

(3) The remaining patients are classified as intermediate risk***.***

The 2003 **Leibovich score** of each patient was calculated according to the original scoring algorithm as below:

| **Feature** | **Score** |
| --- | --- |
| pT stage |  |
| pT1a | 0 |
| pT1b | 2 |
| pT2 | 3 |
| pT3/T4 | 4 |
| pN status |  |
| pN0/Nx | 0 |
| pN1/N2 | 2 |
| Tumor size |  |
| ＜10 cm | 0 |
| ≥ 10 cm | 1 |
| Nuclear grade |  |
| G1-2 | 0 |
| G3 | 1 |
| G4 | 3 |
| Necrosis |  |
| Absent | 0 |
| Present | 1 |

Patients were stratified into low- (with 0 to 2 scores), intermediate- (with 3 to 5 scores), and high-risk (with 6 or more scores) groups, accordingly.

1. ***Supplementary S2. Definitions of various CT phases***

Unenhanced phase (UP) image of each patient was firstly obtained, then, (CMP, 30 s delay), nephrographic phase (NP, 90 s delay), and excretory phase (EP, 10 mins delay) images were acquired orderly after injection of a 90–100 ml volume of iodinated contrast medium (350 mg I/ml) at a rate of 2.5 ml/s.

1. ***Supplementary S3. The specific classifications of extracted radiomics features***

The open source ‘Pyradiomics’ was then used to extract radiomics features, which composing first order features, shape features, gray level co-occurrence matrix (GLCM) features, gray level size zone matrix (GLSZM) features, gray level run length matrix (GLRLM) features, neighbouring gray tone difference matrix (NGTDM) features, and gray level dependence matrix (GLDM) features. In total, 1316 features were extracted.

1. ***Supplementary S4****.* ***Radiomics feature demission reduction procession***

First of all, the extracted radiomics features were standardized separately using z-scores normalization. The average ICC of all extracted radiomics features were 0.87±0.39 and 0.85±0.42 for intra- and inter-observer intraclass correlation analysis, respectively, demonstrating an excellent repeatability of tumor segmentations. 985 radiomics features that with ICCs above 0.80 in both intra- and inter-observer analysis were then fed into Univariate Cox proportional hazards regression. The 517 screened difference features (p<0.05) were further investigated via the Spearman rank correlation test, redundant features (internal linear correlation coefficients > 0.80) were removed (76 features left). Thirdly, a least absolute shrinkage and selection operator (LASSO) Cox regression algorithm with a 10-fold cross-validation was applied to identify the most appropriate subset of prognostic radiomics features.

1. ***Supplementary S5****.* ***The calculation formula of the Rad-Score***

The Rad-score was calculated via combining the selected features and corresponding weights in the multivariate Cox regression analysis.

Rad-Score = -0.02808969 * original_glrlm_ShortRunEmphasis +

0.13760744 * log.sigma.2.0.mm.3D_gldm_LargeDependenceEmphasis +

0.07616516 * wavelet.LHL_glrlm_LongRunEmphasis +

-0.09079299 * wavelet.LHH_glcm_SumEntropy +

0.23679674 * wavelet.HLL_gldm_DependenceNonUniformityNormalized
